# Novel Analysis Method for Beating Cells Videomicroscopy Data: Functional Characterization of Culture Samples

**DOI:** 10.3389/fphys.2022.733706

**Published:** 2022-02-15

**Authors:** Jonathan Béland, James Elber Duverger, Philippe Comtois

**Affiliations:** ^1^Research Centre, Montreal Heart Institute, Montreal, QC, Canada; ^2^Department of Pharmacology and Physiology, Université de Montréal, Montreal, QC, Canada; ^3^Institute of Biomedical Engineering, Université de Montréal, Montreal, QC, Canada

**Keywords:** imaging analysis, contractile activity, cardiomyocyte monolayer, non-linear analysis, heterogeneity, spatial-temporal activity

## Abstract

Cell culture of cardiac tissue analog is becoming increasingly interesting for regenerative medicine (cell therapy and tissue engineering) and is widely used for high throughput cardiotoxicity. As a cost-effective approach to rapidly discard new compounds with high toxicity risks, cardiotoxicity evaluation is firstly done *in vitro* requiring cells/tissue with physiological/pathological characteristics (close to *in vivo* properties). Studying multicellular electrophysiological and contractile properties is needed to assess drug effects. Techniques favoring process automation which could help in simplifying screening drug candidates are thus of central importance. A lot of effort has been made to ameliorate *in vitro* models including several *in vitro* platforms for engineering neonatal rat cardiac tissues. However, most of the initial evaluation is done by studying the rate of activity. In this study, we present new approaches that use the videomicroscopy video of monolayer activity to study contractile properties of beating cells in culture. Two new variables are proposed which are linked to the contraction dynamics and are dependent on the rhythm of activity. Methods for evaluation of regional synchronicity within the image field of view are also presented that can rapidly determine regions with abnormal activity or heterogeneity in contraction dynamics.

## Introduction

Cell culture of cardiac tissue analog is becoming increasingly interesting for regenerative medicine [conditioning of pre-injected stem cell-derived cardiomyocytes (Pillekamp et al., 2012; Hazeltine et al., 2014) and tissue engineering (Vunjak-Novakovic et al., 2011; Lu et al., 2013; Nunes et al., 2013; Wendel et al., 2013; Zhang et al., 2013)] and widely used for high throughput cardiotoxicity evaluation (Dick et al., 2010; Navarrete et al., 2013). Cardiotoxicity is a leading cause of market withdrawal for drugs (Stevens and Baker, 2009; Ferri et al., 2013) some because of inducing cardiac dysfunction. Many drugs demonstrate cardiotoxicity due to chronic exposure to anthracyclines such as doxorubicin (Menna et al., 2008) which are cytotoxic cancer drugs (Schimmel et al., 2004; Yeh et al., 2004). Chronic cardiotoxicity is usually evaluated in animal models including adult rodents (Alderton et al., 1992; Desai et al., 2013) or canine (Herman et al., 1983), over periods of months of exposition. As a cost reduction approach to rapidly discard new compounds with high toxicity, cardiotoxicity evaluation is done *in vitro* with neonatal rat ventricular myocyte cultures and ultimately with human stem cell-derived cardiomyocyte. *In vitro*, spontaneous activity and contraction can be influenced when cardiomyocytes are grown on different surfaces (Engler et al., 2008). As such, studies typically do not extend beyond 10 days (Shirhatti et al., 1986; Dorr et al., 1988), limiting their relevance as models for chronic exposure. It has been suggested that biologically softer material could favor more rhythmic activity (Hazeltine et al., 2014) although it may be material dependent (Boudreau-Beland et al., 2015). Traditional *in vitro* systems also do not recapitulate the native tissue architecture or extracellular microenvironment of the heart, both of which are known to regulate myocyte phenotype (Feinberg et al., 2012; Sheehy et al., 2012). Furthermore, studies with animals and animal-derived cells are not always relevant to humans due to species-dependent differences (Sham et al., 1995), indicating a need to develop *in vitro* systems that are compatible with human-derived cardiac myocytes (Zhang et al., 2009). A lot of effort have been made to ameliorate *in vitro* models including several *in vitro* platforms for engineering neonatal rat cardiac tissues with simultaneous quantification of contractile function in response to variables such as tissue architecture (Grosberg et al., 2011; Feinberg et al., 2012), mechanical stretch (McCain et al., 2013), electrical stimulation (Chiu et al., 2011), or gelatin hydrogels developed as muscular thin film substrates (McCain et al., 2014).

Studying multicellular electrophysiological and contractile properties is needed to assess drug effects and techniques that can favor the automation of the process helping in simplifying the screening process. Techniques for imaging contractile activity has been developed for decades including approaches to measure spontaneous rhythms in culture (Rohr, 1990) to study the rate and stability of activity (Boudreau-Beland et al., 2015). Less costly and more easily distributed imaging chip-scale lensless wide-field-of-view microscopy imaging technique have been proposed which can render microscopy images of growing or confluent cell cultures (Zheng et al., 2011). Long-term culture of engineered animal and human cardiac tissues coupled to less invasive data recording on activity and contractility to better predict adverse or functional effects of drugs on the heart is highly desirable.

In this study, we present new approaches that use the videomicroscopy video to study contractile properties of beating cells in culture. Two new variables are proposed which are linked to the contraction dynamics and are dependent on the rhythms of activity. Methods for evaluation of regional synchronicity within the imagined field of view are also presented that can swiftly determine regions with abnormal activity or heterogeneity in contraction dynamics.

## Materials and Methods

### Cardiomyocyte Isolation Procedure

All animal-handling procedures were concordant with the Canadian Council on Animal Care guidelines and were approved by the institutional Animal Research Ethics Committee. Isolation was performed according to the protocol of the neonatal cardiomyocyte isolation kit from Worthington. In summary, 1–3 days old rats (Sprague-Dawley, Charles River) were sacrificed by decapitation. Beating hearts were removed from the rats and immediately put in cold Ca^2+^ and Mg^2+^-free Hank’s Balanced Salt Solution. The ventricular muscle was selected by excision and the tissue was minced on ice into 1–3 mm^3^ pieces. The mixture was subjected to purified enzymatic digestion (trypsin and collagenase). Isolated cells (enriched cardiomyocytes) were counted and seeded at a density of 10^6^ cells/mL in the seeding area of the membrane pre-coated with 0.2% porcine-derived gelatin (G1890, Sigma) and 0.00125% fibronectin solution (F1141, Sigma). Cells were grown for 24 h in DMEM (319-050-CL, Wisent) with 5% fetal bovine serum (FBS, SH30396.03, Thermo Fisher Scientific Co., Ltd.) and 1% penicillin/streptomycin (450-201-EL, Wisent). Cardiomyocytes were then FBS starved with 1% penicillin/streptomycin in DMEM 24 h prior to the experiments. For the set of experiments used to study the effects of electrically pacing the cardiomyocytes at different pacing cycle length (PCL), the cells were washed and the medium changed to fresh DMEM without FBS just prior to record the activity (thus not being starved for 24 h prior to the experiments).

### β-Adrenergic Stimulation

Acute effects of the β-adrenergic agonist, isoproterenol (ISO, I6504, Sigma-Aldrich) was studied by videomicroscopy with final concentration of 100 nmol/L at 1 min after injection.

### Videomicroscopy Recording

Phase contrast videos of neonatal cardiomyocytes were acquired after 48 h post seeding *in vitro* with a Dalsa HM640 camera (*N*_*y*_ = 640 × *N*_*x*_ = 480 pixels) at rates of 30, 50, or 100 frame per second (fps) coupled to an inverted Nikon optical microscope (10× magnification). The field of view (FOV) covered by the camera was 0.44 mm by 0.33 mm.

### Mathematical Models of Cardiomyocyte Electrophysiology and Contraction

The Morotti et al. (2014) model of mouse ventricular myocyte modified to integrate the Negroni et al. (2015) model of myofilament contraction as used by Surdo et al. (2017)^1^ was simulated at PCL ranging from 200 to 1,000 ms to study the effects of period of activity. To account for the duration of transient solution when starting from the resting steady-state, simulation output at 30 and 60 s are considered in this study. The Negroni et al. (2015) of rabbit ventricular myocyte was also simulated at different PCL for a 30 s duration of simulation. A modified version of the rabbit model was also studied where two parameters of the myofilament section were multiplied by a factor of 5: Yb (binding rate of Ca^2+^) and *f* (transition rate between the 1st and 2nd state of the troponin system). The parameter Yv (part of the detachment function) was scaled down by a factor of 5.

The models were numerically integrated in Matlab (R2008, MathWorks Inc., Natick, MA) using the variable-step, variable-order solver based on the numerical differentiation formulas of orders 1–5 for stiff system (ode15s). Relative error tolerance was set to 10^–5^.

### Statistical Analysis

All statistical analyses have been done in R (version 3.1.3). Linear regression was used to test for variable dependency on period of activity. The analysis of covariance (ANCOVA) was used to compare group and covariate effects. Means were compared with Student’s *t*-test.

## Analysis Approach and Results

### Studying the Global Contraction Characteristics

The proposed initial approach is using the raw video signal and calculating for each pixel the difference between frames separated by a delay corresponding to a defined frame interval.

**FIGURE 1:**
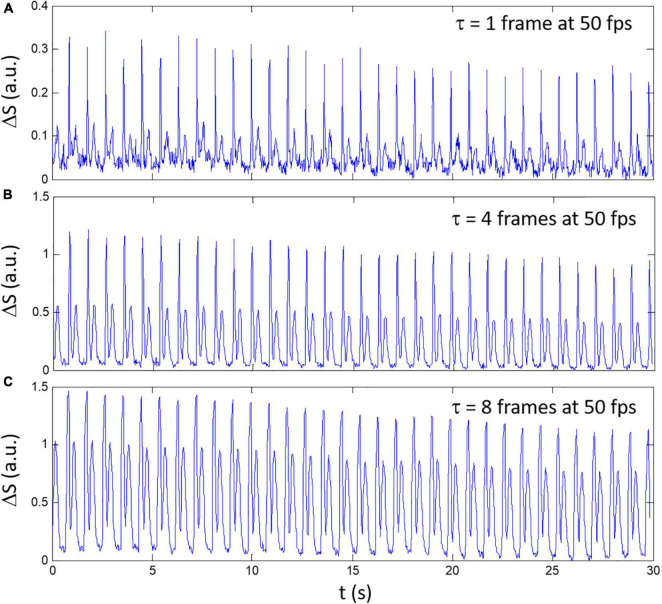
An example of composite signals Δ*S* obtained with different delay between frames for a spontaneously beating sample. **(A)** τ = 1 frame, **(B)** τ = 4 frames, and **(C)** τ = 8 frames. The video was recorded at 50 fps.

Let *M*(*t*) be the global camera frame recorded at time *t*. The composite signal Δ*S* is calculated using the following Equation:


(1) $$\Delta S\left(t\right)=\frac{1}{N_{x}N_{y}}\sum_{j=1}^{N_{y}}\sum_{i=1}^{N_{x}}\left|\frac{M_{i,j}\left(t\right)-M_{i,j}\left(t-\tau \right)}{\tau }\right|,$$

where τ is a discrete time delay (or number of frames for delay multiplied by the time between frames Δt = 1/fps), *N*_*y*_ and *N*_*x*_ are, respectively, the number of horizontal and vertical pixels such that *i* and *j* are the pixels coordinates along the horizontal and vertical axis. An example of the composite signal ΔS for a spontaneously beating sample is shown in Figure 1 for different delay (τ = 1,4, and 8 multiplied by Δ*t*). The shortest delay τ = Δ*t* corresponding to a frame-by-frame difference shows the highest noise level (Figure 1A) while increasing τ to 4Δ*t* (Figure 1B) and 8Δt (Figure 1C) decreased the amplitude difference between the high and low amplitude peaks.

### Link Between Videomicroscopy Signal and Cellular Contraction

**FIGURE 2:**
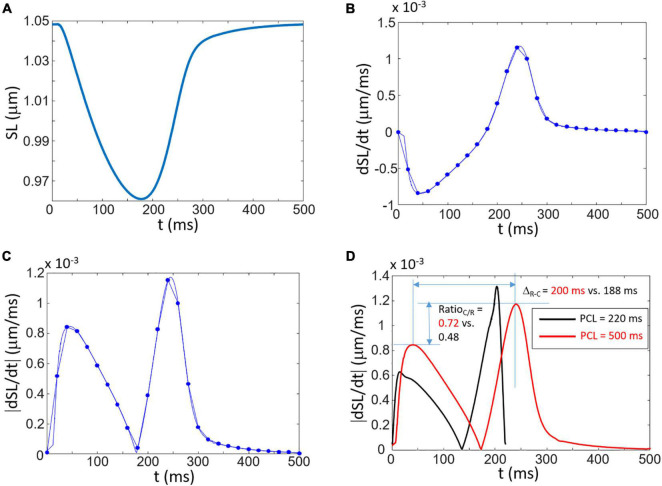
Simulation of contraction and the link to the composite signal Δ*S.* **(A)** Shortening of the sarcomere length (SL) as a function of time. **(B)** The time derivative of SL. The dots correspond to the derivative calculated with a sub-sample signal corresponding to 50 samples/s (Δt = 0.02 s). **(C)** Rectified signal of **(B)** showing double peaks corresponding to the contraction and relaxation phases. **(D)** Explanation of two specific measures (Δ_*R*–*C*_ and Ratio_*C/R*_) obtained from simulations at two difference PCL (black curve: PCL = 220 ms; red curve: PCL = 500 ms). A shorter PCL leads to a decreased Δ_*R*–*C*_ and decreased Ratio_*C/R*_.

**FIGURE 3:**
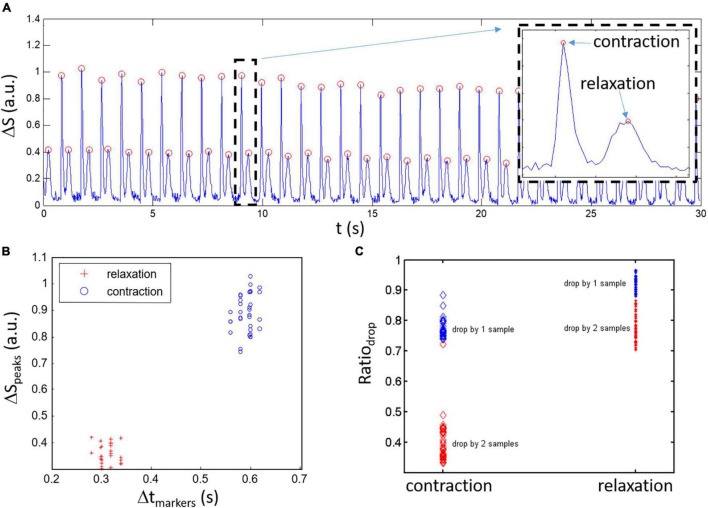
Classification of ΔS peaks for a spontaneously beating sample. **(A)** An example of composite signal ΔS with detected peaks (red circles) showing alternation between high amplitude and low amplitude peaks. Inset: blow up of the activity within the dashed black rectangle which highlights the high amplitude contraction peaks followed by the lower amplitude relaxation peak like the derivative of the simulated contraction model. **(B)** Contraction and relaxation clusters are relatively well separated when viewed in the space with *x*-axis being the time between markers and *y*-axis being the peak amplitude (Δ*S*_*peaks*_). **(C)** Estimation of the change in ΔS around the peaks given by the Ratio_*drop*_ (amplitude of 1st and 2nd samples around the peaks divided by the peak amplitude). Lower ratio Ratio_*drop*_ is linked to faster change in Δ*S* around the peaks for the contraction peaks compared to the relaxation.

*In silico* data and analysis reveal that the composite signal Δ*S* calculated from Eq. (1) can be interpreted as follow. The absolute derivative of the time-dependent cell length calculated from simulated sarcomere length (SL) obtained from the simulation is given by:


(2) $$dSL_{abs}\left(t\right)=\left|\frac{dSL}{dt}\right|$$

where SL is the sarcomere length. The change in SL calculated with the Surdo et al. (2017) model paced at a PCL of 500 ms is presented in Figure 2A. The time derivative of this contraction signal is depicted in Figure 2B which shows the initial contraction (negative derivative) followed by the positive relaxation signal. As shown in Figure 2C, there is a clear similarity between the rectified derivative given by Eq. (2) and the videomicroscopy signal obtained with Eq. (1) as shown in Figures 1A, 3A (highlighted in the inset of panel A).

Two specific measures are proposed that are presented in Figure 2D which corresponds to the amplitude ratio between the contraction and relaxation peaks (Ratio_*C/R*_) and the time between the contraction and relaxation peaks (Δ_*R*–*C*_). The effects of the PCL on contraction signal and specific measures are shown in Figure 2D which highlights the sensitivity of the measures on rhythm and calcium dynamics. Decreasing the PCL from 500 to 220 ms lead to a decrease of 6% of Δ_*R*–*C*_ and of 33% of Ratio_*C/R*_.

Automatic discrimination between contraction and relaxation peaks is important in the perspective of user-friendliness and for the approach to have a clear potential in a high throughput screening/testing system. The approach is based on the observations of experimental data showing differences in amplitude and rate of decrease around the peaks between the contraction and relaxation. Starting from a ΔS signal as shown in Figure 3A, three conditions are being used to detect and validate classification of the peaks: 1- there is alternance between contraction and relaxation peaks, 2- clusters are usually separated in the variable space given by the time difference between peaks (Δt_*markers*_) and amplitude of the peaks (ΔS_*peaks*_) as depicted in Figure 3B, 3- experimental data has sharpest peaks for contraction and widest peaks for relaxation resulting in more rapid amplitude loss around the maximum ΔS peak amplitude. The last condition can be easily evaluated by taking the amplitude for 1 sample (first sample before and after the position of the peak) and 2 samples (second sample before and after the peak) around the peak divided by the peak amplitude (Ratio_*drop*_: the ratios given by the value of the first and second samples around the peak divided by the value of the signal for the peak define). As such, Ratio_*drop*_ serves to evaluate how fast the amplitude decreases around the peak distinguishing fast changes (narrow contraction peaks)_and slower changes (wide relaxation peaks). The obtained data are presented in Figure 3C where a lower average Ratio_*drop*_ is found for the contraction peaks (left circles; 0.77 ± 0.03 and 0.40 ± 0.06 n.u.) compared to the relaxation peaks (right dots; 0.92 ± 0.02 and 0.78 ± 0.04 n.u.). Using the second neighboring points (what we labeled drop by two samples) helps separating the contraction and relaxation groups of points (the red points behind more clearly separated as a function of Ratio_*drop*_).

### Changes in Contraction Measures

**FIGURE 4:**
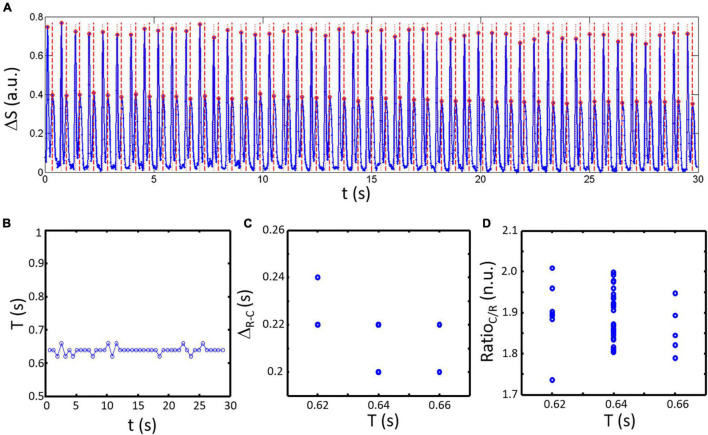
Temporal variation in spontaneous activity obtained by analysis of ΔS with 50 fps. **(A)** The composite signal obtained for a representative sample (blue line). Detected peaks are highlighted by red circles. Post classification results are shown by dotted red lines (contraction peaks) and dashed red lines (relaxation peaks). **(B)** The period of activity given the time difference between contraction peaks shows a constant period of activity equal to the pacing cycle length (PCL). **(C)** Calculated Δ_*R*–*C*_ as a function of the period of activity (corresponding here to the time elapse since the last contraction) is only varying by ± one sample. **(D)** Ratio_*C/R*_ is more varying compared to Δ_*R*–*C*_.

An example of an analyzed acquisition is presented in Figure 4. The signal ΔS is shown in Figure 4A with detected peaks (red circles) and resulting classification highlighted for contraction (red dotted lines) and relaxation peaks (red dashed lines). The resulting series of spontaneous activity shows a constant period T (Figure 4B: 0.64 ± 0.01 s) almost no variation in Δ_*R*–*C*_ (Figure 4C: 0.22 ± 0.01 s). However, Ratio_*C/R*_ shows greater variability between samples (Figure 4C: 1.89 ± 0.06 n.u.) mainly due to variations in the maximum amplitude of the contraction peaks as seen on the signal in Figure 4A.

**FIGURE 5:**
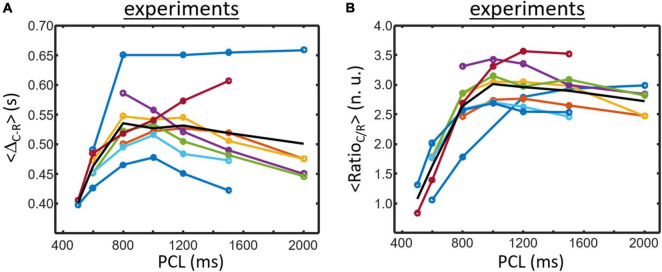
Experimental data obtained for 8 samples electrically paced at different PCL. Each colored line corresponds to a sample results except for the black line that represent the mean of the 8 culture samples. **(A)** Δ_*R*–*C*_ is generally increasing for small PCL. The average curve (black line) exhibits a biphasic morphology which initially increases followed by a small decrease for large PCL (5/8 samples have similar morphology). **(B)** Ratio_*C/R*_ usually starts low and is increasing when PCL increases. A biphasic pattern is also found for Ratio_*C/R*_ (7/8 samples) and is also seen for the average curve (black line).

A set of 8 samples data obtained at PCL ranging from min. 500 to max. 2,000 ms are presented in Figure 5 (each color corresponding to a given cell culture sample except for the black line that corresponds to the mean value of the samples). <Δ_*R*–*C*_> (Figure 5A) is the lowest for shortest cycle length and is augmenting with PCL increasing except for one sample. The average curve (black line) shows a slight biphasic shape with intermediate PCL values being slightly higher than for the highest PCL (6/8 samples clearly showing a biphasic shape).

Data for <Ratio_*C/R*_> are presented in Figure 5B. The lowest value is usually found for lower PCL and is increasing with PCL increasing (7/8 samples). There is again a tendency for a biphasic shape of the curves which is more visible for the average curve (black line).

### Comparison With Simulation Data

**FIGURE 6:**
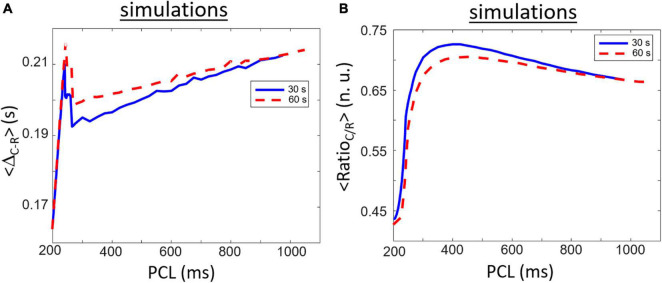
Similar to Figure 5 but for simulation results obtained after 30 (blue line) and 60 s (red line) of simulation. **(A)** Rapidly increasing Δ_*R*–*C*_ for short PCL increase followed be a rapid decrease and a long and slow increase when increasing the PCL. **(B)** Ratio_*C/R*_ shows a clear biphasic waveform starting with a small value for short PCL which rapidly increases to a maximum value for PCL around 420 ms followed by a slower decrease while PCL increases. The maximum value of Ratio_*C/R*_ is smaller at 60 s compared to 30 s indicating the part of the biphasic shape is due to transient dynamics.

Long-term simulations of the Surdo et al. (2017) mouse ventricular model ionic model have been done for a comparison purpose with the experimental data. Simulation results for <Δ_*R*–*C*_> are shown in Figure 6A. The general shape of the curves differs between the experiments and simulations. Similar to experimental data, simulated <Δ_*R*–*C*_> is the lowest for shortest cycle length and is augmenting with PCL increasing for shortest PCL. There is, however, a particular biphasic shape (more like a narrow peak) as the maximum value is found around 240–245 ms before rapidly decreasing followed by a slow increase with PCL increasing. This narrow peak comes from the morphology of the |dSL/dt| relaxation phase which has a double hump shape. The peak in the <Δ_*R*–*C*_> as a function of PCL comes from a change from the first hump to be higher to the second bump thus resulting from an added delay in the marker for relaxation (∼0.02 s). The maximum (experiments: 0.54 s; simulation: 0.21 s) and range (experiments: 0.14 s; simulation: 0.05 s) of <Δ_*R*–*C*_> is also less than in experiments. We compared the results for two duration of simulation (30 and 60 s of simulation for different constant PCL) to highlight the possible transient behavior of the model. The rate main difference between shorter (30 s) and longer (60 s) simulations are a higher <Δ_*R*–*C*_> right after the peak resulting in a slower increase with increasing PCL for PCL > 300 ms.

Simulated data for <Ratio_*C/R*_> are presented in Figure 6B. Similar to experimental data, the lowest value is found for lower PCL and is increasing with PCL increasing. There is a clear biphasic shape of the curve that seems also to be found in some experimental samples in Figure 5B. The highest values in simulated <Ratio_*C/R*_> is found near PCL = ∼375 ms. The biphasic shape comes from the difference in rate of change between the contraction and relaxation peaks with increasing PCL. Starting at PCL = 360 ms, the rate of increase in the amplitude of the relaxation peak as a function of increasing PCL is greater than the rate of increase of the contraction peak. As such, the Ratio_C/R for PCL > 360 ms is decreasing with PCL increasing. There is, however, a decrease in highest <Ratio_*C/R*_> for longer simulations (60 s compared to 30 s) indicating that it is, at least in part, linked to transient behavior of the model. More importantly is the range of values which differs from the experimental data. <Ratio_*C/R*_> is always less than one for simulated data with the mouse model while experimental data are mostly greater than one (except for some cases at lowest PCL). The differences are important because value lower than one means that the rate of relaxation is faster than the rate of contraction.

The differences between the experimental and simulated data lead us to test if it was because of the ionic model. Thus, the rabbit ventricular model by Negroni et al. (2015) was simulated with normal parameter values (ctl) and with two contraction parameters (modified, see “Materials and Methods” section for details). The change in parameters (between ctl and modified models) did not significantly impact the action potential duration when paced at 500 ms as shown in Supplementary Figure 1A. While the ctl model has a relaxation rate higher than the contraction rate (black curve in Supplementary Figure 1C) resulting in a Ratio_*C/R*_ < 1, the modified model (green curve) shows a faster contraction than relaxation (Ratio_*C/R*_> 1). A visual comparison of the curves clearly shows that the modified model has longer Δ_*R*–*C*_ compared to the ctl model. <Δ_*R*–*C*_> and <Ratio_*C/R*_> as a function of PCL for the Negroni et al. (2015) model can be found in Supplementary Figures 2A,B, respectively. The ctl rabbit ventricular model shows an increase in <Δ_*R*–*C*_> compared to Morotti et al. mouse model (maximum value around 0.25 s vs. ∼0.21 s). Interestingly, simulating the modified Negroni et al. (2015) rabbit model resulted in even longer <Δ_*R*–*C*_> (∼0.35 s) getting closer to the experimental values. While <Ratio_*C/R*_> for the ctl Negroni et al. (2015) rabbit ventricular model is always less than 1 the modified version resulted in <Ratio_*C/R*_>> over almost all the PCL range.

### Average Values of Samples in Spontaneous Activity

**FIGURE 7:**
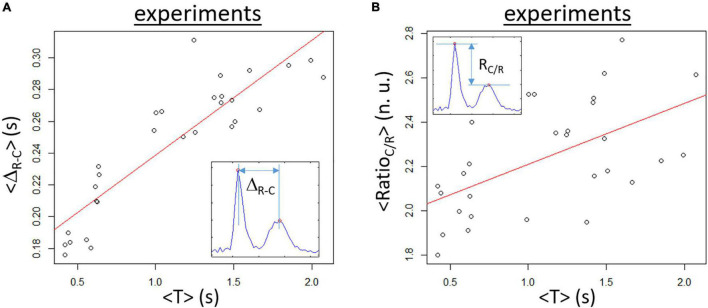
Experimental data obtained for samples in spontaneous rhythm. **(A)** Mean temporal Δ_*R*–*C*_ (<Δ_*R*–*C*_>) is increasing when the mean T (<T>) is augmenting. The red line corresponds to the linear regression fit (slope of 0.072, *p* < 0.001, corr. = 0.89). **(B)** Mean Ratio_*C/R*_ (<Ratio_*C/R*_>) as a function of <T> showing a slight augmentation with increasing <T>. The linear regression line (red line) has a slope of 0.276 (*p* < 0.01, corr = 0.56).

A set of samples (*n* = 29) has been analyzed. For each video of 30 s duration, the average values <T>, <Δ_*R*–*C*_>, and <Ratio_*C/R*_> were calculated as the average of the temporal values obtained from the signal analysis of individual acquisition. A clear monotone increase in <Δ_*R*–*C*_> with increasing <T> is found as depicted in Figure 7A with ∼40% change between the minimum and maximum values. The linear regression (*p* < 0.001) has a slope of 0.072 s/s (represented as a red line) and intercept of 0.166 s. As expected, the variability is greater for <Ratio_*C/R*_> but a trend to an increasing ratio as <T> augments is found. However, the variation with the period is less with ∼23% change between the minimum and maximum values in the dataset (see Figure 7B). The fitted regression line (*p* < 0.005, red line on the panel) has a slope of 0.276 s^–1^ and an intercept of 1.93.

### Influence of β–Adrenergic Stimulation on <Δ_*R*–*C*_> and <Ratio_*C/R*_>

**FIGURE 8:**
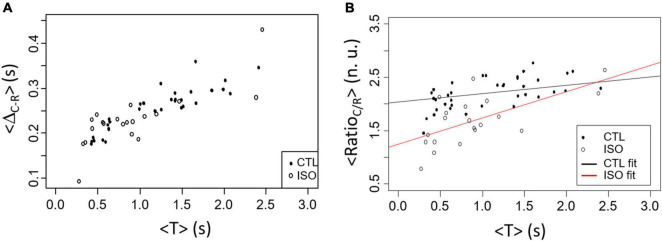
Comparison between CTL (black points) and ISO (white circles) contraction results in spontaneous rhythm. **(A)** <Δ_*R*–*C*_> as a function of <T> does not shows dissimilarity between groups. **(B)** <Ratio_*C/R*_> as a function of <T> differs between CTL and ISO with both a significant <T> and treatment effects (CTL vs. ISO).

The variation in the contraction measures that can be evaluated by our videomicroscopy approach has been tested with isoproterenol (ISO), a β–adrenergic agonist. Results are presented in Figure 8. As expected, the period of activity is significantly decreased with ISO compared to CTL (0.9 ± 0.6 s vs. 1.9 ± 2.4 s in CTL, *p* < 0.05). Similar variation of <Δ_*R*–*C*_> is found between CTL and ISO groups as a function of <T> although <T> is in average smaller with ISO as expected (Figure 8A). Interestingly the <Ratio_*C/R*_> showed statistically significant <T> and group effects with *p* < 0.001. The slope of the linear regression being larger for the ISO group compared to CTL (0.146 for CTL vs. 0.490 for ISO).

### Could the Proposed Measures, Δ_*R*–*C*_ and Ratio_*C/R*_, Be Useful for Cardiotoxicity Testing?

In order to see if Δ_*R*–*C*_ and Ratio_*C/R*_ may be interesting to evaluate cardiotoxicity, we used the change in sarcomere length data presented in Figure 5B in the study by Timolati et al. (2009) comparing the rat ventricular cardiomyocyte contraction paced with a period of 500 ms in control and treated with 1 μmol/L of doxorubicin for 48 h. The data was digitized and sampled with 10 ms between samples (shown in Supplementary Figure 3). Using the same approach as depicted in Figure 2 for the Surdo et al. (2017) ionic model, we calculated the mean of the last two contractions (skipping the first one) yielding Δ_*R*–*C*_ = 0.11 s in control compared to 0.07 s for the doxorubicin-treated cardiomyocyte. Ratio_*C/R*_ was increased in cardiomyocyte treated with doxorubicin (1.3 in control vs. 1.5 for the doxorubicin-treated cardiomyocyte). Although this result is very limited, it points to the interest of the measures in detecting contraction changes and evaluating cardiotoxicity.

### From Global to Regional Analysis

All the previous analyses presented in this study are based on a global composite signal calculated using the entire FOV. The same approach can be used for sub-regions of the FOV by calculating a composite signal for each section. We present here two additional approaches aiming to study spatial-temporal differences in videomicroscopy signals.

#### Finding Areas With Non-correlating Activity in Spontaneous Activity

The first approach is based on determining how local signal correlates with the global composite signal. The first step is to split the image in contiguous regions of *N_*sub*,x_* and *N*_*sub,y*_ pixels. The local composite signals are calculated over all sub-regions of *N*_*sub,x*_ and *N*_*sub,y*_ pixels from the total FOV with Eq. (3) (similar to Eq. 1).


(3) $$\begin{array}{c} \Delta S_{x^{\prime },y^{\prime }}\left(t\right)=\frac{1}{N_{sub,y}N_{sub,x}}\sum_{u=1}^{N_{sub,y}}\sum_{v=1}^{N_{sub,x}}\\ \;\left|\frac{\begin{array}{c} M_{u+N_{sub.y}\left(y^{\prime }-1\right),v+N_{sub.x}\left(x^{\prime }-1\right)}\left(t\right)\\ -M_{u+N_{sub.y}\left(y^{\prime }-1\right),v+N_{sub.x}\left(x^{\prime }-1\right)}\left(t-\tau \right) \end{array}}{\tau }\right| \end{array}$$

where *N_*sub*,y_* = *N*_*sub,x*_ = 10, *M*(*t*) is the movie frame at time *t*, *u* and *v* are the local coordinate within each sub-region of the FOV and *x*′ and *y*′ are the new position within the segmented FOV (such that *x*′ = 1, *y*′ = 1 is the first pixel of the new video calculated from on the individual signals of the pixels *y* = 1–10 and *x* = 1–10 from the original video).

**FIGURE 9:**
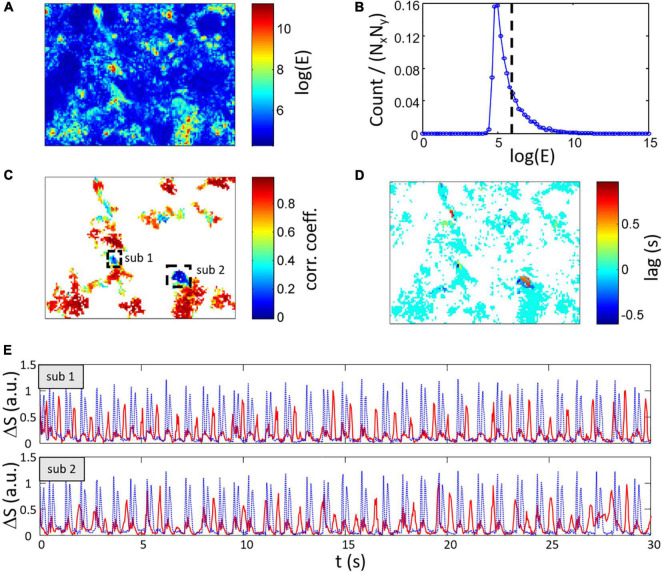
Estimation of heterogeneous spontaneous contraction rhythms within the FOV. **(A)** Map of log(E) with the energy E calculated from Eq. (4) showing area with no contraction signal in blue. **(B)** Histogram of log(E) with the dashed line representing the threshold [log(E) > 6] use to select high energy signal regions. **(C)** Maximum correlation coefficient and **(D)** lag between local Δ*S_*x*′,y′_*(*t*) and global Δ*S*(*t*) for regions having log(E) > 6. Two low CC regions labeled sub 1 and sub 2 are highlighted. Most of the regions show high CC with 0 s lag. **(E)** Signal from region sub 1 (top axes) and sub 2 (bottom axes) showing the low CC (shown as red lines vs. global Δ*S* as dotted blue lines) and global Δ*S*.

The energy (*E*) of the local composite signals is calculated using:


(4) $$E_{x^{\prime },y^{\prime }}=\frac{1}{N_{t}-\tau }\sum_{t=\tau }^{N_{t}}{\left(\frac{d\Delta S_{x^{\prime },y^{\prime }}\left(t\right)}{dt}\right)}^{2}$$

where *N*_*t*_ is the number of frames of the original video. An example of the spatial distribution of log(*E_*x*′,y′_*) is displayed in Figure 9A and the corresponding histogram can be found in Figure 9B. High energy regions of the FOV are selected using a thresholding approach.

The correlation coefficient (CC) and lag between the global signal Δ*S*(*t*) and local composite signals Δ*S_*x*′,y′_*(*t*) are calculated. An example of the resulting map of coefficients and lag are respectively depicted in Figures 9C,D after keeping pixels with log(E) > 6. In this example, most of the relevant section of the FOV have a correlation coefficient greater than 0.8 and a lag of 0 s which indicates that the local activity is highly similar between these regions and the global activity. However, some regions show lower correlation including regions with a correlation value of less than 0.4 (regions labeled sub 1 and 2 in Figure 9C). Interestingly, these regions have also non-zero lags. Using a thresholding approach on the correlation coefficient map, two corresponding clusters of low correlation with high energy can be detected and the average signal from these clusters are shown in Figure 9E. The signals in both sub 1 and 2 regions (red line) have strong peaks usually not occurring simultaneously with the global composite signal (blue dotted line). Please note that lower amplitude peaks are also found in this signals that correlate with the global activity.

#### Digging Deeper in Acquisitions With Complex Global Signal

**FIGURE 10:**
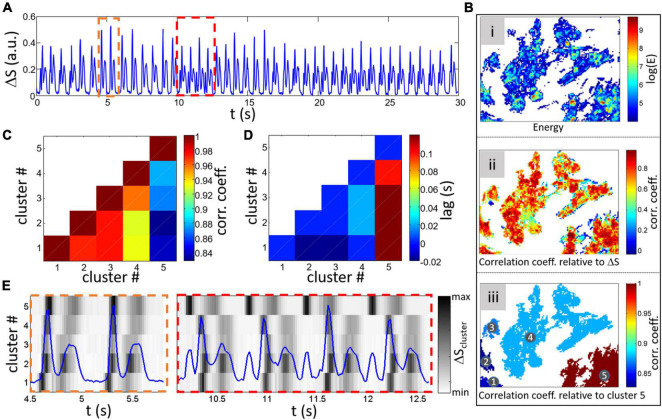
Analysis of a different global ΔS for a different sample with complex activity. **(A)** Example of a non-stationary global signal with time intervals where multiple peaks (different to the contraction/relaxation peaks usually found). Two intervals are highlighted by dashed rectangles with the orange one showing the normal double peak signal and the red rectangle with multiple peaks. **(Bi)** Map of log(E) after thresholding [log(E) > 4]. **(Bii)** CC between individual cluster signals and global Δ*S*. **(Biii)** Labeled separated clusters with colorscale representing the CC between cluster 5 and other clusters. **(C)** Matrix of CC between clusters (Pillekamp et al., 2012; Lu et al., 2013; Wendel et al., 2013; Zhang et al., 2013; Hazeltine et al., 2014) with diagonal equal to 1 and **(D)** lag for maximum CC. Minimum CC and maximum lag are found between cluster 5 and all 4 other clusters. **(E)** Cluster Δ*S* signals for the 5 within the two intervals highlighted by rectangles in **(A)** is shown by a grayscale (peaks are darker and lower values whiter). The global Δ*S* is plotted in blue on top for comparison.

Conditions that alter the development and function of cultured monolayers can affect the spatial-temporal activity. Confluent monolayers usually show consistent and relatively stationary signal with the common contraction/relaxation peaks as shown in Figure 3A. However, more complex global composite signals can be found such as multiple peak complexes as can be seen in the example shown in Figure 10A.

To investigate the causes of these complex patterns, a thresholding on the energy was done as previously showed [map of log(*E*) is shown in Figure 10Bi after thresholding with log(E) > 4]. The method is based on cross correlation between two time series shifted relatively in time (the time shift being the lag). Calculation of the correlation coefficient with the global composite signals does not highlight important regions with low coefficient (Figure 10Bii). However, calculating the correlation coefficient and lag between cluster signals [from the average Δ*S_*x*′,y′_*(*t*) of each cluster] results in the matrix plot shown in Figures 10C (correlation coefficient) and D (lag). Both panels show a clear difference in correlation and lag between cluster 5 and the others (labeling of the clusters can be found on the map showing the correlation coefficient between cluster 5 and others in Figure 10Biii).

The causes of the correlation differences can be investigated by further studying the differences in the cluster Δ*S_*x*′,y′_*(*t*) signals represented by a grayscale in Figure 10E. A closer look at the maps shows that synchronization between cluster 5 and the others varies over time with almost simultaneous activity within the orange dashed rectangle (which has a corresponding normal double peak feature in the global signal shown by the blue line). However, the complex multi-peaks section encompassed within the red dashed rectangle corresponds to an earlier contraction in cluster 5 (with delays between cluster 4 and 5 of 100 and 160 ms for the 2nd and 3rd beats in the rectangle). The non-stationary aspect of the global signal can thus be understood by a change in timing of cluster 5 activity in respect to other clusters where the abnormal added peaks are found when a long delay between cluster contractions occurs. Depending on signal quality, the contraction/relaxation analysis approach presented in the first part of this article could be done on cluster signals to extract and compare temporal activity variations.

## Discussion and Conclusion

In this study, we proposed novel approaches to study beating dynamics in cell culture. To our knowledge, the main contractility analysis method described herein the first to study the contractility characteristics based on monolayer videomicroscopy data. Indeed, previous methods focused mainly on period/frequency of activity (Rohr, 1990; Kim et al., 2011). Based on a simple composite signal calculated as the variation in pixels intensity, two main variables can be determined: the time difference and the ratio between the contraction and relaxation peaks. These new variables could be interesting to determine toxic effects on cardiomyocytes more importantly regarding heart failure risk. Both variables showed to be dependent on the period of activity (either paced as shown in Figure 5 or spontaneously beating as in Figure 7). A note that a direct comparison between the spontaneously beating data and paced data cannot be done as being two different sets of experiments with different FBS-starved prior to experiments.

Comparison with simulation results show qualitative similarities between simulation and experimental data. However, there are quantitative differences on both variables of interest as, for example, Ratio_*C/R*_ is less than one in the simulations but not in experiments. A recent study clearly showed that the rate of contraction is faster than the rate of relaxation (which would lead to Ratio_*C/R*_> 1) for both atrial and ventricular adult rat cardiomyocytes (Nollet et al., 2020). Visual inspection of published mouse cardiomyocyte shortening seems to also point to a Ratio_*C/R*_> 1 (Lim et al., 2000). There is of course a correlation between the calcium dynamic and contraction characteristics. An interesting example has been published by del Monte et al. (1999) in their study on contractile function in isolated cardiomyocytes from failing human hearts. Failing heart cardiomyocytes are known to have decreased expression and function of SERCA2. The data presented show that the contraction is much slower than relaxation when looking at the cell shortening. As such, we expect to have a RatioC/R less than 1. Overexpression of SERCA2 yielded reverted the cell shortening to a cell shortening similar to the non-failing cardiomyocyte. The mathematical modeling in our study assumed an isotonic contraction while a confluent monolayer should probably be a mixed condition between isometric (on a stiff cell culture substrate) and isotonic on the free top side of the cells. The study by Rodriguez et al. (2011) clearly showed that even isolated (not being a monolayer), the velocity of contraction measured from displacement of an elastic post show a fastest contraction compared to relaxation. It is interesting to note that the force of contraction of an isolated but attached neonatal rat cardiomyocytes is heterogeneous with the highest forces being found at the periphery of the cell and lowest at the center (where the nucleus is found). As such, it is highly probable that the formation monolayer, by having cardiomyocytes constraining each other, would have an impact of the proposed measures. Thus, although there may still be differences between monolayers vs. isolated cells, it is likely that it is not the cause for the contraction rate being smaller than the relaxation rate in the simulations resulting in a Ratio_*C/R*_ smaller than 1. Also, it is highly probable that differences between culture conditions (substrate rigidity, microstructure for cardiomyocyte alignment, mechanical and electrical stimulation) known for influencing cardiomyocyte function would also influence the contraction function and thus, possibly change the measures in some way. The exact link between the contraction waveform and the actual change in contraction measures obtained from the composite signal requires further investigation.

It is interesting and promising to see that the proposed measures is dependent on the rate of activity. We have shown that adrenergic stimulation (with isoproterenol) which is well known for its isotropic effect results in a change in Ratio_*C/R*_ confirming that the proposed measures are of interest to evaluate contraction changes by pharmacological agents. Although it remains to be tested experimentally, analysis of previously published contraction changes induced by doxorubicin resulted in variations of both Ratio_*C/R*_ and Δ_*R*–*C*_. The last two proposed approaches (Figures 9, 10) are interesting to evaluate the homogeneity of the monolayer activity and, in case of heterogeneous dynamics, quantify the correlation between monolayer areas. Conditions inducing partial electrical decoupling could be detected.

The proposed approaches may be also interesting in limiting impact on the beating cells as proposed previously (Kim et al., 2011; Pushkarsky et al., 2014) which remains to be evaluated. Light impact on cellular process can be decreased by limiting exposure and careful selection of wavelength bands to favor contrast but it is believed to be minimal (Rohr, 1990). Although extension to lens-free CMOS imaging remains to be tested, our method can be adjusted to cover various scales of field of view. The limited time resolution due to the relatively low frame per second from a reasonably priced sensor could be a limitation. However, even a slow frame rate of 30 fps (as used for the CTL vs. ISO experiments) showed a significant difference in the proposed contraction measures. Recent development in imaging technology and communication hardware with off-the-shelf USB 3.0 or MIPI CSI-2 cameras open the way to greater than 100 fps simple sensor solution. Even the Raspberry Pi camera module can now easily allow up to 90 fps with VGA resolution (640 × 480 pixels) opening the way to simple low-cost high throughput parallel screening.

The proposed alternative methods described here that aim to study heterogeneity in contraction signal are interesting as they can estimate cell culture characteristics impossible to study directly with classical methods (Rohr, 1990; Kim et al., 2011). Here, detection of localized abnormal activity (compared to the global activity) could also be a measure of cell/tissue sample deterioration. Moreover, the change in synchronization between regions, an important variable that can be link to intercellular coupling and be a factor favoring arrhythmia, can be evaluated. As such, actual application of these approaches and evaluation of their relevance as appropriate biomarker of new drug cardiotoxicity could be of great interest.

## Data Availability Statement

The raw data supporting the conclusions of this article will be made available by the authors, without undue reservation.

## Ethics Statement

The animal study was reviewed and approved by the Montreal Heart Institute Animal Research Ethics Committee, Montreal Heart Institute.

## Author Contributions

JB collected the data. JB and PC helped design the initial phase of the study, did experimental data analysis and interpretation, and drafted the article. JD and PC did mathematical modeling data collection, analysis, and interpretation. JB, JD, and PC gave final approval of the version to be published.

## Conflict of Interest

The authors declare that the research was conducted in the absence of any commercial or financial relationships that could be construed as a potential conflict of interest.

## Publisher’s Note

All claims expressed in this article are solely those of the authors and do not necessarily represent those of their affiliated organizations, or those of the publisher, the editors and the reviewers. Any product that may be evaluated in this article, or claim that may be made by its manufacturer, is not guaranteed or endorsed by the publisher.

## Footnotes

1
https://somapp.ucdmc.ucdavis.edu/Pharmacology/bers/
