# Supplementary material for: Novel Analysis Method for Beating Cells Videomicroscopy Data: Functional Characterization of Culture Samples
Source: Front Physiol. 2022 Feb 15;13:733706. doi: 10.3389/fphys.2022.733706 (PMC8886216; doi:10.3389/fphys.2022.733706)
Supplement: Supplementary file 1 [file Image_1.pdf]

Supplementary figures

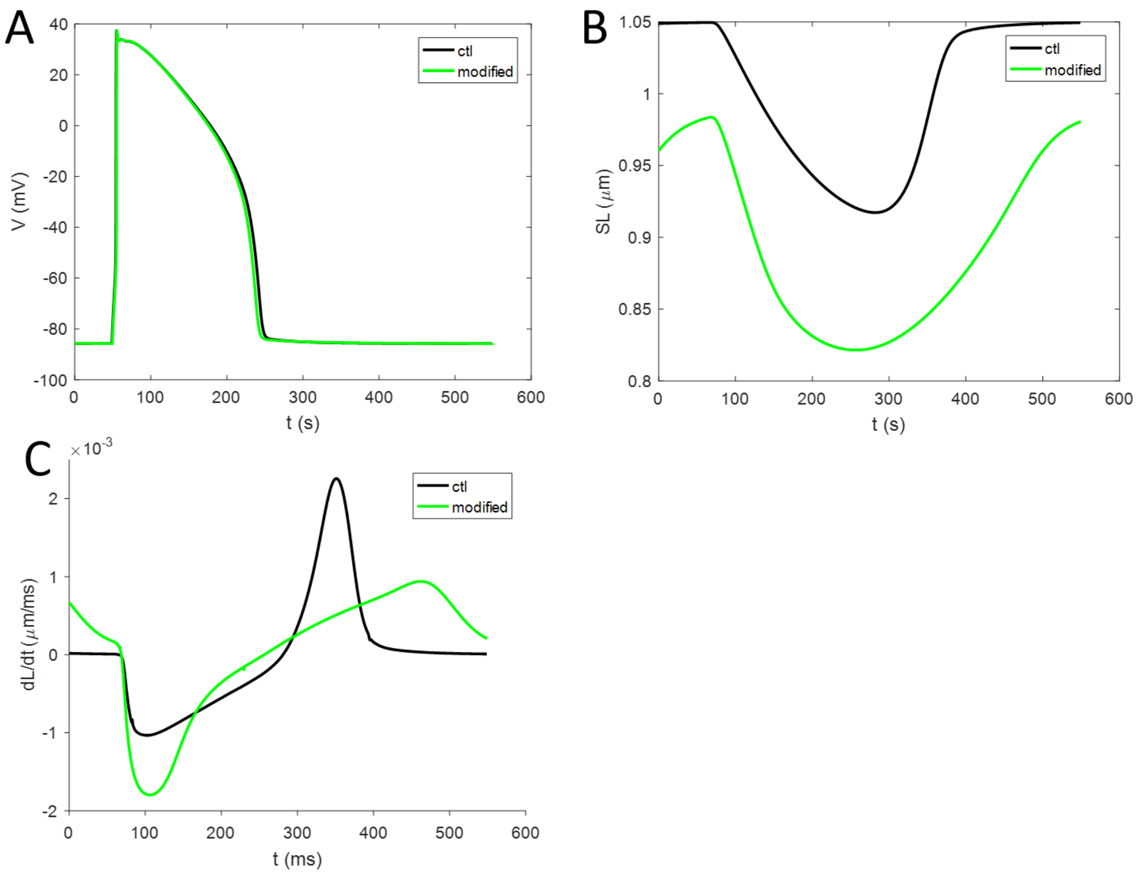

Figure S1 Simulation results obtained with the Negroni et al. rabbit ventricular model (1) with a PCL of 500 ms. Two versions of the model was simulated: control (ctl) and a modified version where the parameters f and Yb were multiplied by a factor of 5 (modified). A). Action potentials showing a limited decreased in duration for the modified model. B). Sarcomere length (SL) changes and C) Rate of change of SL.

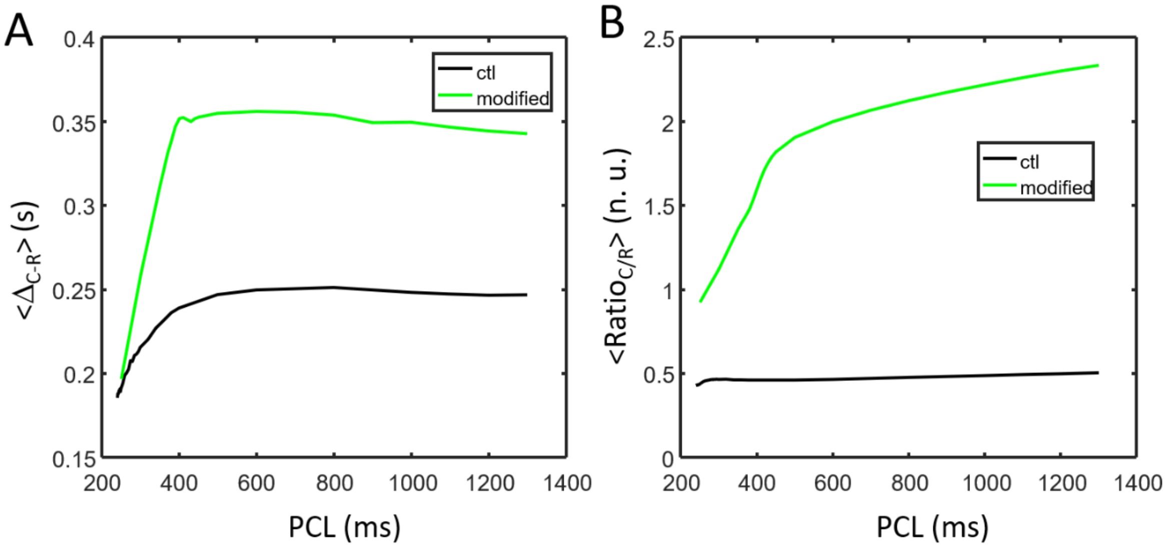

Figure S2 A) Δ_{R-C} as a function of PCL for the Negroni et al. rabbit ventricular model (1) (ctl) and modified as detailed in Fig. S1 caption. B) $Ratio_{C/R}$ as a function of PCL.

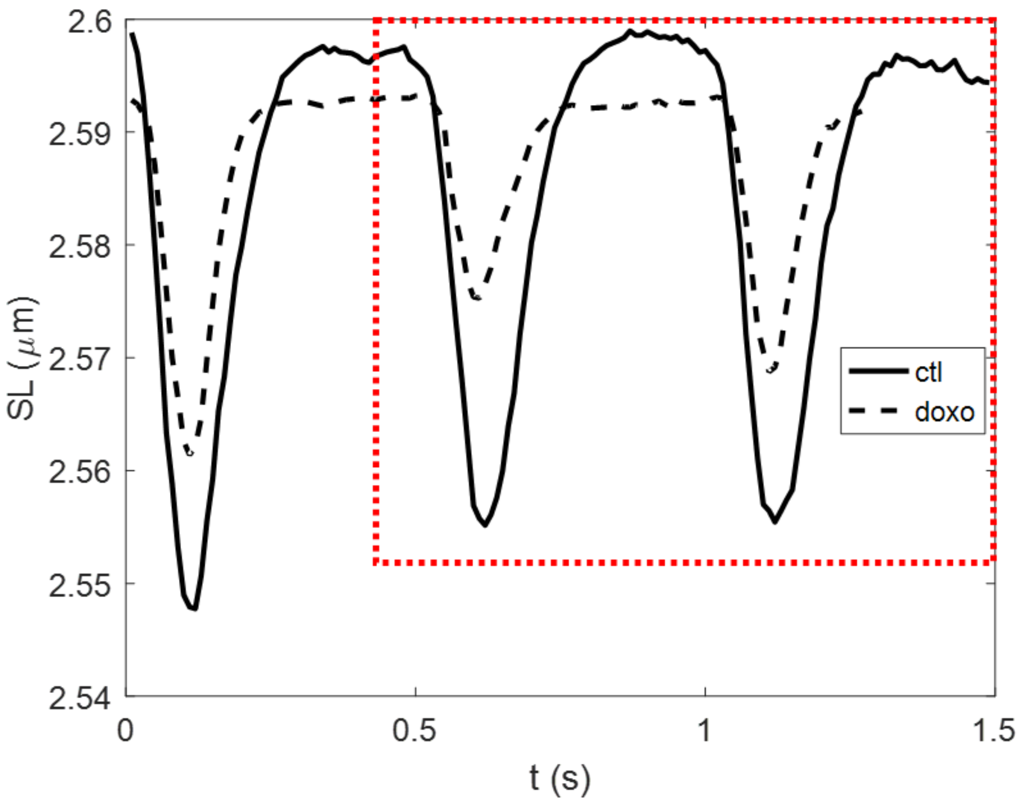

Figure S3 Digitized sarcomere length (SL) data obtained from Fig. 5B in the study by Timolati et al (2) comparing the rat ventricular cardiomyocyte contraction paced with a period of 500 ms in control and treated with 1 μmol/L of doxorubicin for 48 h. The data was digitized and sampled with 10 ms between samples. The last two beats (highlighted by the dotted red box) were used to calculate the Δ_{R-C} and $Ratio_{C/R}$.

References

1. Negroni JA, Morotti S, Lascano EC, Gomes AV, Grandi E, Puglisi JL, et al. beta-adrenergic effects on cardiac myofilaments and contraction in an integrated rabbit ventricular myocyte model. J Mol Cell Cardiol. 2015;81:162-75.
2. Timolati F, Anliker T, Groppalli V, Perriard JC, Eppenberger HM, Suter TM, et al. The role of cell death and myofibrillar damage in contractile dysfunction of long-term cultured adult cardiomyocytes exposed to doxorubicin. Cytotechnology. 2009;61(1-2):25-36.
